# Exo-erythrocytic development of two *Haemoproteus* species (Haemosporida, Haemoproteidae), with description of *Haemoproteus dumbbellus*, a new blood parasite of bunting birds (Emberizidae)

**DOI:** 10.1016/j.ijpara.2023.02.009

**Published:** 2023-05-30

**Authors:** Mélanie Duc, Tanja Himmel, Mikas Ilgūnas, Vytautas Eigirdas, Herbert Weissenböck, Gediminas Valkiūnas

**Affiliations:** a Nature Research Centre, Akademijos 2, 08412 Vilnius, Lithuania; b Institute of Pathology, Department for Pathobiology, University of Veterinary Medicine Vienna, Veterinaerplatz 1, 1210 Vienna, Austria; c Ventės Ragas Ornithological Station, Marių 24, 99361 Ventė, Lithuania

**Keywords:** *Haemoproteus*, *Haemoproteus dumbbellus* n. sp., Meronts, Megalomeronts, Avian haemosporidians, Chromogenic *in situ* hybridization

## Abstract

Avian haemosporidians are widespread parasites categorized into four families of the order Haemosporida (Apicomplexa). Species of the subgenus *Parahaemoproteus* (genus *Haemoproteus)* belong to the Haemoproteidae and are transmitted by *Culicoides* biting midges. Reports of death due to tissue damage during haemoproteosis in non-adapted birds have raised concerns about these pathogens, especially as their exo-erythrocytic development is known for only a few *Haemoproteus* spp. More research is needed to better understand the patterns of the parasites’ development in tissues and their impact on avian hosts. Yellowhammers *Emberiza citrinella* (Emberizidae) and common house martins *Delichon urbicum* (Hirundinidae) were screened for *Haemoproteus* parasites by microscopic examination of blood films and PCR-based testing. Individuals with single infection were selected for histological investigations. H & E-stained sections were screened for detection and characterization of the exo-erythrocytic stages, while chromogenic *in situ* hybridization (CISH) and phylogenetic analysis were performed to confirm the *Haemoproteus* origin and their phylogenetic relationships. *Haemoproteus dumbbellus* n. sp. was discovered in *Emberiza citrinella* single-infected with the lineage hEMCIR01. Meronts of *H. dumbbellus* n. sp. developed in various organs of five of six tested individuals, a pattern which was reported in other *Haemoproteus* species clustering in the same clade, suggesting this could be a phylogenetic trait. By contrast, in *Delichon urbicum* infected with the *Haemoproteus* lineage hDELURB2, which was linked to the more distantly related parasite *Haemoproteus hirundinis*, only megalomeronts were found in the pectoral muscles of two of six infected individuals. All exo-erythrocytic stages were confirmed to be *Haemoproteus* parasites by CISH using a *Haemoproteus* genus-specific probe. While the development of meronts seems to be typical for species of the clade containing *H. dumbbellus*, further investigations and data from more species are needed to explore whether a phylogenetic pattern occurs in meront or megalomeront formation.

## 1 Introduction

Genetic lineages of avian haemosporidians (Haemosporida, Apicomplexa) have been recorded in 2195 bird species all over the world, except for Antarctica (MalAvi database, Lund University, Lund, Sweden, https://130.235.244.92/Malavi/ last access in September 2022) (Bensch et al., 2009; Clark et al., 2014). The parasites classify into four families – Plasmodiidae, Haemoproteidae, Leucocytozoidae and Garniidae (Valkiūnas, 2005). Parasites of the Haemoproteidae belong to the genus *Haemoproteus*, which consists of two subgenera – *Haemoproteus* and *Parahaemoproteus –*, species of which are transmitted by louse flies (Hippoboscidae) and biting midges (Ceratopogonidae, subgenus *Culicoides*), respectively (Valkiūnas, 2005; Chagas et al., 2019; Valkiūnas and Atkinson, 2020). After injection of sporozoites by the vectors into susceptible avian hosts, exo-erythrocytic stages develop (Valkiūnas and Iezhova, 2017). Within these stages, merozoites form, which later infect host erythrocytes to become gametocytes, the infective stage for vectors (Valkiūnas, 2005).

The Haemoproteidae family is rich in species number, with 177 *Haemoproteus* spp. described based on the gametocytes’ morphology and peculiarities of their influence on host cells (Valkiūnas and Iezhova, 2022). At the same time, 1905 genetic lineages of these parasites have been reported, but only 159 (or 8%) of them were linked to 76 morphospecies (MalAvi database, last access in September 2022 (Bensch et al., 2009)), suggesting an even higher species diversity. Opposed to the rapidly increasing data on the genetic diversity of these parasites due to the implementation of PCR-based methodologies, many aspects about the development of these parasites in their avian hosts remain unknown for most of the described species. This is particularly true for the exo-erythrocytic stages of the parasites, which are known only fragmentarily for less than 30 species of *Haemoproteus* (Valkiūnas and Iezhova, 2017; Himmel et al., 2019, 2021; Ilgūnas et al., 2019, 2022; Ortiz-Catedral et al., 2019; Duc et al., 2020, 2021; Hernández-Lara et al., 2021; Yoshimoto et al., 2021; Harl et al., 2022).

Exo-erythrocytic stages (or tissue stages) of *Haemoproteus* parasites did not attract much attention until the recent reports of deaths due to haemoproteosis (Ortiz-Catedral et al., 2019) in non-adapted birds, which usually do not contract the parasites of certain lineages and thus are less able to cope with them. Such infections often result in severe disease and even mortality due to damage of organs by the parasites’ exo-erythrocytic stages (Ortiz-Catedral et al., 2019).

*Haemoproteus* parasites can develop into two different types of exo-erythrocytic stages – meronts and megalomeronts. Meronts usually do not exceed 70 μm in length (Hernández-Lara et al., 2021); they are often of irregular form and covered by a thin eosinophilic wall. Megalomeronts have been reported to be up to 800 μm in size (Duc et al., 2021); they are often of roundish or oval form and covered by a thick capsular-like wall of host origin. Recent studies on exo-erythrocytic stages of *Haemoproteus* spp. reported the presence of either only meronts (*Haemoproteus attenuatus* (Hernández-Lara et al., 2021)) or only megalomeronts (*Haemoproteus minutus, Haemoproteus asymmetricus, Haemoproteus majoris, Haemoproteus syrnii, Haemoproteus* sp. (Himmel et al., 2019; Ilgūnas et al., 2019, 2022; Ortiz-Catedral et al., 2019; Yoshimoto et al., 2021)) in the investigated avian hosts. Among these parasites, only two species were reported to develop tissue stages in more than one host species, i. e. *H. syrnii* megalomeronts in two host species *(Strix aluco* and *Strix uralensis* (Ilgūnas et al., 2022)), and *H. majoris* megalomeronts in three host species *(Parus major, Turdus pilaris*, and *Phylloscopus sibilatrix* (Ilgūnas et al., 2019; Duc et al., 2020)). In older literature, four *Haemoproteus* species – *Haemoproteus passeris*, *Haemoproteus handai*, *Haemoproteus mansoni* and *Haemoproteus columbae* – were reported to develop both types of exo-erythrocytic stages, albeit not in the same host individuals and without molecular support (G. Valkiūnas, pers. comm.) (Peirce, 1976; Burtikashvili, 1978; Miltgen et al., 1981; Atkinson et al., 1986, 1988; Earle et al., 1993; Peirce et al., 2004; Valkiūnas and Iezhova, 2017). Unfortunately, experimental studies of the exo-erythrocytic development of *Haemoproteus* parasites are scarce (Atkinson et al., 1986, 1988). Due to the limited information on the merogonic development for the majority of described *Haemoproteus* spp., it remains elusive whether the formation of meronts and megalomeronts reflect functional or developmental adaptations or represent phylogenetically informative traits of the parasites on species levels. These data are necessary to better understand the patterns of development of tissue stages in *Haemoproteus* spp. and to explore whether it is possible to predict the exo-erythrocytic development of closely related parasites using phylogenetic approaches. This, in turn, could help to predict in which organs and how tissue stages could develop in natural and non-adapted hosts during haemoproteosis and then, possibly to plan research on the development of treatment measures.

The present study aimed to investigate the exo-erythrocytic merogony of two *Haemoproteus* parasite lineages, for which tissue stages have not been described: hEMCIR01, a lineage commonly found in *Emberiza citrinella* (MalAvi database, last access in September 2022, (Bensch et al., 2009)) but not yet attributed to morphospecies, and hDELURB2, a putative lineage of *Haemoproteus hirundinis* based on high similarity with *H. hirundinis* hDELURB1 (Chagas et al., 2019) and often detected in *Delichon urbicum* (MalAvi database, last access in September 2022, (Bensch et al., 2009)). Samples collected from *E. citrinella* and *D. urbicum* were investigated for single infections with hEMCIR01 and hDELURB2, respectively, and the development of their exo-erythrocytic stages were examined and discussed in regard to their phylogenetic relationship with other avian haemoproteids. Furthermore, blood stages of these parasites were morphologically characterized, and the lineages attributed to their morphospecies.

## 2 Materials and methods

### 2.1 Sample collection

Yellowhammers *E. citrinella* and common house martins *D. urbicum* were collected in the ornithological station Ventė Cape (55°20′38.93″N, 21°11′34.05″E, see https://www.vros.lt), Lithuania, in May of 2017, 2018, 2019, and 2021, using mist nets and a large stationary trap (‘Rybachy’ type trap). Blood samples were obtained from the brachial vein. From each bird, blood films were prepared, and approximately 20 μL of blood was collected in SET buffer (0.05 M Tris, 0.15 M NaCl, 0.5 M EDTA, pH = 8.0) and stored at −20 °C. The blood films were fixed in methanol, stained with Giemsa and screened microscopically for the presence of haemosporidian parasites using a standard protocol for the detection of avian haemosporidians (Valkiūnas, 2005). Six birds of each species with single infections of *Haemoproteus* parasites as determined by blood film examination, were euthanized, and their organs (brain, heart, lungs, trachea, oesophagus, gizzard, intestine, liver, spleen, kidneys, reproductive organs, and pectoral and leg muscles) were collected for histological examination. In addition to the *E. citrinella* collected in Lithuania, tissue samples of two *E. citrinella* were retrieved from the archive of the Institute of Pathology at the University of Veterinary Medicine Vienna, Austria. These two birds were submitted for post-mortem examination in August 2004, and their organs collected and fixed in neutral buffered formalin for histological examination. Samples of the brain, liver, and spleen were frozen and stored at −20 °C for molecular analysis. Blood films were not available for these two individual specimens.

### 2.2 Parasitaemia, prevalence and parasite morphology

Blood films were screened using a BX41 (Olympus, Tokyo, Japan) light microscope to determine the infection status of the collected birds and the prevalence of the parasite (percentage of birds infected with the target parasites species out of all collected individuals of the same host species). Blood films were analyzed for 15–20 min at medium magnification (400×) followed by examination of 100 microscope fields at high magnification (1000×). For dissected individuals with available blood films, the intensity of parasitaemia was determined by counting the number of parasites per 1000 erythrocytes or per 10,000 erythrocytes in case of low parasitaemia (Godfrey et al., 1987); measurements and images of gametocytes were taken using an Olympus DP12 digital camera and the Olympus DP-SOFT software. The standard range of *Haemoproteus* parasite characters were used for morphological characterization of gametocytes and their host cells for a new *Haemoproteus* sp. (Valkiūnas, 2005).

### 2.3 Histology and chromogenic in situ hybridization (CISH)

Tissue samples were fixed in 10% neutral buffered formalin, dehydrated in increasing ethanol concentrations (70–100%), clarified in xylene, and embedded in paraffin wax. Histological sections of 2–3 μm were prepared from all collected organs, stained with haematoxylin & eosin staining (H & E), and screened for exo-erythrocytic stages at magnifications of 100x and 200x using Olympus BX41 and B51 light microscopes equipped with Olympus DP12 or UC90 digital cameras, respectively (Olympus, Tokyo, Japan). Higher magnifications (400×, 1000×) were used for taking pictures of exo-erythrocytic stages, using the Olympus image softwares DP-SOFT or cellSens Entry. Acquired photographs were adjusted for brightness and contrast and assembled in CorelDraw 2019 (RRID:SCR_014235, https://www.coreldraw.com/en/). Based on the photographs, measurements of exo-erythrocytic stages were taken using the ImageJ-based imaging processing package Fiji (Image J 1.53c, National Institutes of Health, Bethesda, MD, USA, downloaded at https://imagej.net/software/fiji accessed on August 24 2022) (Schindelin et al., 2012).

In parallel to H & E-stained histological preparations, chromogenic *in situ* hybridization (CISH) using a *Haemoproteus* genus-specific probe (Haemo18S) targeting the *18S* ribosomal RNA of the parasites (Himmel et al., 2019) was conducted on at least one section per organ per individual according to previously described protocols (Dinhopl et al., 2011; Himmel et al., 2019). A sample of one *E. citrinella* individual from Vienna included in this study was previously used for testing the specificity and sensitivity of the Haemo18S probe (Himmel et al., 2019).

### 2.4 DNA extractions, PCRs, and sequencing

For molecular characterization of parasites detected in the blood films, DNA extraction and PCR screening were performed. DNA extractions of the SET buffer-stored blood samples were done following the ammonium acetate protocol (Richardson et al., 2001). DNA extractions of frozen tissue samples of two *E. citrinella* were performed using the DNeasy Blood & Tissue Kit (QIAGEN, Venlo, Netherlands) following the manufacturer’s protocol with the modification of performing two elution steps, each with 100 μl instead of a single elution. The second eluate was used for PCR.

Molecular screening for avian haemosporidians was performed using a nested PCR protocol, which amplifies a 478 bp section of the cytochrome b (*cytb*) gene (Bensch et al., 2000; Hellgren et al., 2004). The outer primer pair HAEMNFI/HAEMNR3, and the inner primer pairs HAEMF/HAEMR2 and HAEMFL/HAEMR2L were applied to detect *Haemoproteus* and *Plasmodium*, and *Leucocytozoon* parasites, respectively. Amplification of parasite DNA using the protocol of Hellgren et al., (2004) was unsuccessful in four of six *D. urbicum* samples, so they were additionally screened with the nested PCR protocol described in (Beadell et al., 2004; Hellgren et al., 2004; Duval et al., 2007; Pérez-Rodríguez et al., 2013) using the primer pairs PLAS1F/HAEMNR3 and 3760F/HAEMJR4, which detect parasites from all three genera. PCR profiles for both nested PCRs were kept as per original protocols, including negative (distilled water) and positive (previously determined *Plasmodium/Leucocytozoon* infected sample) controls. PCR products were examined on 2% agarose gels. Successfully amplified fragments were prepared for Sanger bi-directional sequencing with a Big Dye Terminator V3.1 Cycle Sequencing Kit and ABI PRISM™ 3100 capillary sequencing robot (Applied Biosystems, Foster City, CA, USA), or were sent for bi-directional sequencing to Microsynth (Microsynth, Vienna, Austria).

Obtained sequence chromatograms were analysed using the softwares Geneious Prime 2022.0.2 (Dotmatics, Auckland, New Zealand, https://www.geneious.com), and Bioedit (https://bioedit.software.informer.com/) (Hall, 1999). All sequences were subjected to BLAST search in the MalAvi database, last access in April 2022 (Bensch et al., 2009) and NCBI GenBank (National Library of Medicine, Bethesda, Maryland, https://www.ncbi.nlm.nih.gov/genbank/, last access in April 2022) to identify detected lineages.

### 2.5 Phylogenetic analysis

A Bayesian phylogenetic tree was calculated using only lineages which were identified to species level, including 34 *Haemoproteus* lineages from the subgenus *Parahaemoproteus*, three lineages from the subgenus *Haemoproteus*, and one lineage of *Leucocytozoon*, which was used as outgroup. All sequences were retrieved from the GenBank database, using their common lineage names from the MalAvi database. The model GTR+I+G was used after checking for the best-fit model in jModeltest-2.1.10 (Guindon and Gascuel, 2003, Darriba et al., 2012) with AIC, AICc, BIC and DT. MrBayes plugin v3.2.6 (Huelsenbeck and Ronquist, 2001) was run in Geneious for 5 million generations, sampled every 100th generation, and discarding the first 25% trees as a ‘burn-in’ period for the consensus tree.

## 3 Results

### 3.1 Parasite identifications and parasitaemia

Six *D. urbicum* and six *E. citrinella* showed single *Haemoproteus* infections both by microscopic examination and PCR-based testing (Table 1).

**Fig. 1:**
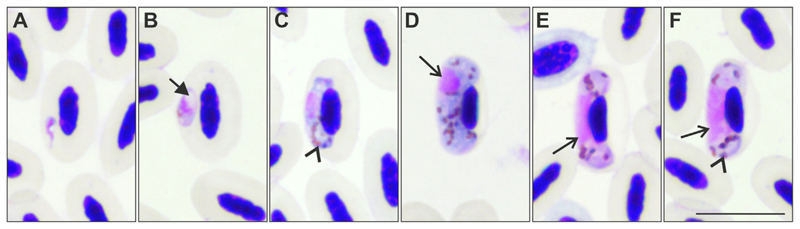
Gametocytes of *Haemoproteus hirundinis* (lineage hDELURB2) from the blood of a common house martin *Delichon urbicum.* Developmental stages are (A, B) young gametocytes (Note the presence of a vacuole in young gametocytes in (B) as indicated by the arrow); (C) growing, and (D) fully grown macrogametocytes; (E) growing, and (F) fully grown microgametocytes. Parasite nuclei in D-F are indicated by arrows. The arrowheads indicate pigment granules. Scale-bar = 10 μm.

Gametocytes found in *D. urbicum* displayed characteristics of *H. hirundinis* (Fig. 1A-F; see description below), and the intensity of parasitaemia in *H. hirundinis*-infected birds ranged from 0.04% to 0.53% (Table 1).

**Fig. 2:**
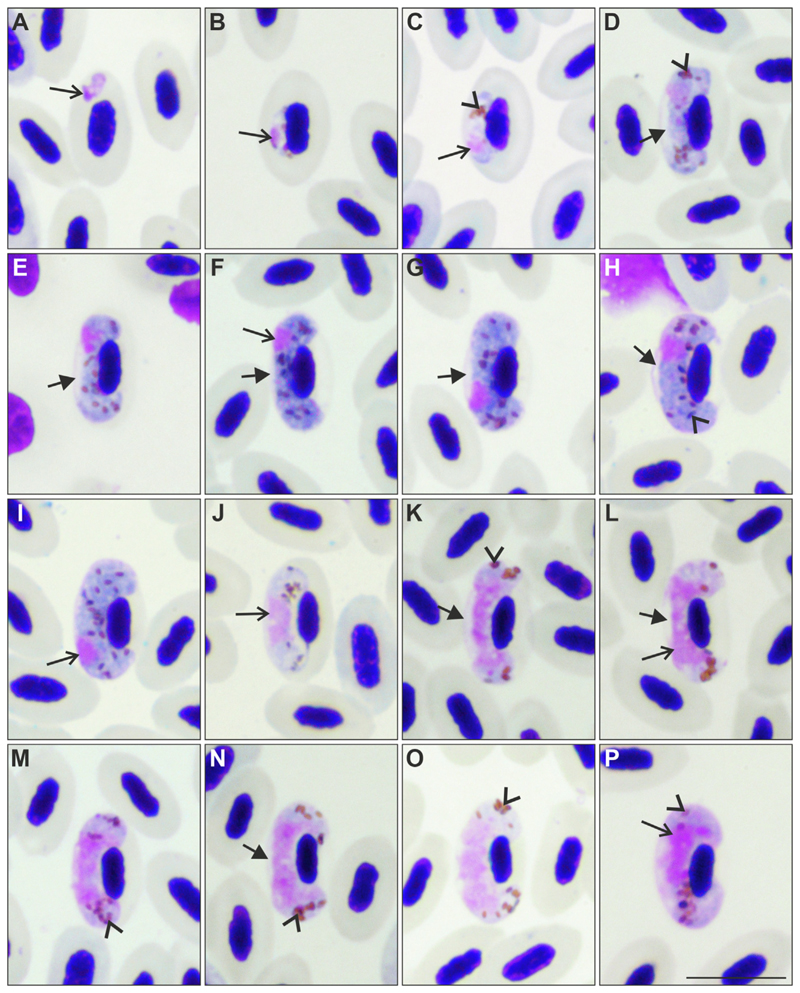
Gametocytes of *Haemoproteus dumbbellus* n. sp. (lineage hEMCIR01) from the blood of a yellowhammer *Emberiza citrinella.* Developmental stages are (A-C) young gametocytes; (D-I) macrogametocytes; (J-P) microgametocytes. The following forms can be distinguished among them: (D, E) growing macrogametocytes, (F, G) advanced macrogametocytes, (H, I) fully grown macrogametocytes, (J, K) growing microgametoccytes, (L-M) advanced microgametocytes, and (N-P) fully grown microgametocytes. Note that early gametocytes (A) do not adhere to the erythrocyte nuclei, but all other blood stages (B-P) adhere to them. The long arrow indicates the parasite nucleus and the arrowheads indicate pigment granules. An unfilled space (indicated by the short arrow in D-H, K, L, N) is present between gametocytes and the envelope of erythrocytes from the stage of developing gametocytes to the stage of fully grown gametocytes. This gives the parasite a dumbbell-like shape at most stages of growth, which is a characteristic feature of this parasite species. Note that this space often maintains in fully grown gametocytes (H, N), a rare character in *Haemoproteus* species. Fully grown gametocytes fill erythrocytes till the poles; they enclose the nuclei of erythrocytes with their ends but do not encircle them completely (H, I, O, P). The macrogametocyte nucleus is subterminal in position; it does not adhere to the erythrocyte nucleus (F-I). Scale-bar = 10 μm.

Gametocytes present in the blood films of *E. citrinella* displayed unique characters which correspond to a new species (Fig. 2A-P) and were described below. The parasitaemia ranged from 0.26% to 1.04% in different infected individuals of *E. citrinella* (Table 1).

### 3.2 Lineages and phylogenetic analysis

Molecular analysis of the partial *cytb* sequences revealed single infections for all 14 individuals investigated. *Delichon urbicum* were all infected with the *Haemoproteus* lineage hDELURB2, and *E. citrinella* were all infected with the lineage hEMCIR01 (Table 1). It is important to note, that the lineage hDELURB2 was amplified in only two of six *D. urbicum* samples using the primers HAEMNFI/HAEMNR3-HAEMF/HAEMR2, while the primers PLAS1F/HAEMNR3-3760F/HAEMJR4 successfully amplified hDELURB2 in all six samples. All sequences were deposited in GenBank under the accession numbers **OQ361943-OQ361953, MN025423, MK330150**, and **MK330152**.

**Fig. 3:**
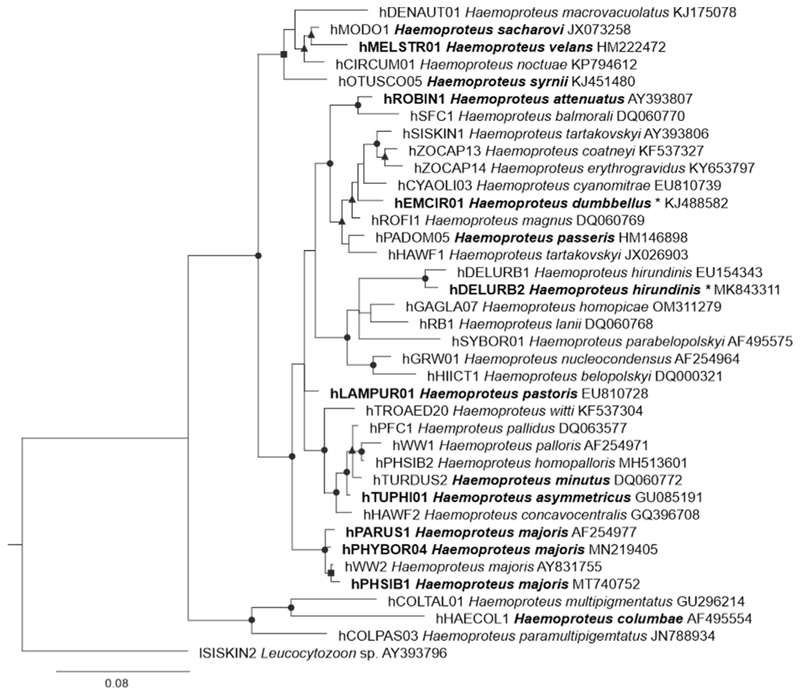
Bayesian phylogenetic tree of partial cytochrome *b* sequences of 37 *Haemoproteus* spp. lineages and one *Leucocytozoon* sp. lineage as outgroup. Parasites were represented by MalAvi lineage names (Bensch et al., 2009), followed by their species names and GenBank sequences accession numbers. Bold font indicates species (and lineage when known) for which exo-erythrocytic stages (meronts or megalomeronts) have been described. Parasites from this study indicated with an asterisk. Posterior probabilities (PP) are provided with symbols: triangles, PP 0.7–0.8; squares, PP 0.8–0.9; and circles, PP 0.9–1.

Phylogenetically, hDELURB2 was closely associated with the *H. hirundinis* lineage hDELURB1, differing by 7 bp or 1.5% in the barcoding region, while hEMCIR01 clustered with *Haemoproteu* ssp. (hROFI1, 9 bp difference), *Haemoproteus tartakovskyi* (hSISKIN1, 13 bp difference), *Haemoproteus cyanomitrae* (hCYAOLI03, 13 bp difference), and *H. passeris* (hPADOM05, 12 bp difference), among others (Fig. 3). With regard to the species for which exo-erythrocytic stages have been described (presented in bold letters in the tree), the lineage hEMCIR01 was most closely associated with *H. attenuatus* and *H. passeris*, both of which were reported to form meronts (Peirce, 1976, Hernández-Lara et al., 2021). *Haemoproteus hirundinis* hDELUBR2 clustered with species for which exo-erythrocytic stages have not been described yet (e. g. *Haemoproteus lanii, Haemoproteus homopicae*, and others) (Fig. 3).

### 3.3 Description of Haemoproteus dumbbellus (lineage hEMCIR01)

*Haemoproteus (Parahaemoproteus) dumbbellus* n. sp.

Type host: Yellowhammer *E. citrinella* (Passeriformes, Emberizidae).

Barcoding DNA sequence: Mitochondrial *cytb* lineage hEMCIR01 (478 bp, GenBank accession numbers **OQ361943-OQ361948**, **MK330150**, **MK330152**).

Additional hosts: the barcoding lineage was recorded in three bird species: *Emberiza cirlus* (Emberizidae), *Phylloscopus trochilus* (Phylloscopidae) and *Sula nebouxii* (Sulidae) (Dimitrov et al., 2010; Levin et al., 2011; Mata et al., 2015; Ellis et al., 2020); however, these studies do not mention the presence of gametocyte stage in these avian hosts.

Type locality: Ventė Ragas Ornithological station, Lithuania (55°20′38.93″N, 21°11′34.05″E).

Site of infection: Gametocytes develop in mature erythrocytes. Exo-erythrocytic stages (meronts) were seen in the lungs, heart, leg muscle, brain, liver, and gizzard of the host.

Prevalence: Eight of 18 individuals examined (44%).

Type specimens: Hapantotypes (gametocytes, *E. citrinella*, sampled in 2018.05.16, 2021.05.17, 2021.05.19 Ventė Cape, Lithuania, coll. Mikas Ilgūnas, accessions nos. 49446NS, 49460NS, 49469NS; exo-erythrocytic stages: 2004.07.30, Güssing, Burgenland, Austria, coll. Tanja Himmel, accessions nos. 49454NS – 49457NS; 2021.05.17, 2021.05.19, Ventė Cape, Lithuania, coll. Mélanie Duc, accessions nos. 49462NS, 49463NS, 49471NS – 49476NS) were deposited to the Nature Research Centre (NRC), Vilnius, Lithuania.

Parahapantotypes (gametocytes, *E. citrinella*, 2018.05.16, 2021.05.17, 2021.05.19, Vente Cape, Lithuania, coll. Mikas Ilgūnas, accessions nos. 49447NS, 49461NS, 49470NS; exo-erythrocytic stages: 2004.07.09, 2004.07.30, Güssing, Burgenland, Austria, coll. Tanja Himmel, accessions nos. 49452NS, 49453NS, 49458NS, 49459NS) were deposited to the NRC. Parahapantotypes (gametocytes, *E. citrinella*, 2021.05.17, 2021.05.19, Ventė Cape, Lithuania, coll. Mikas Ilgūnas, accessions nos. G466262, G466267, G466273; exo-erythrocytic stages: 2004.07.09, Güssing, Burgenland, Austria, coll. Tanja Himmel, accessions nos. G466263 – G466266, 2021.05.17, Ventė Cape, Lithuania, coll. Mélanie Duc, accessions nos. G466268 – G466272) were deposited to the Queensland Museum, Queensland, Australia.

Additional material: Voucher preparations from *E. citrinella* sampled at the type locality*:* gametocytes (accession nos. 49450NS, 49451NS) and histological sections (accession nos. 49464NS – 49468NS, 49477NS – 49480NS) were deposited to the NRC.

Etymology: The species name derives from the English word “dumbbell”. It reflects the dumbbell-like shape of advanced gametocytes due to the presence of a readily visible space between the gametocytes and envelope of infected erythrocytes (Fig. 2D-I). The dumbbell-like form is present at most stages of gametocyte growth, including the fully grown gametocytes – a rare character in avian haemoproteids.

Young gametocytes (Fig. 2A-C): Earliest forms were usually located in a subterminal to terminal position in the infected erythrocytes. They do not adhere to the host cell nuclei, nor to the erythrocyte envelope (Fig. 2A). As the parasite develops, gametocytes closely adhere to the nuclei of erythrocytes at their lateral side and extend longitudinally along the nuclei, but the young growing forms still do not adhere to the erythrocyte envelope (Fig. 2B, C). The gametocyte cytoplasm stains unevenly (Fig. 2C); nuclei are prominent and of irregular shape; pigment granules are well-visible and usually grouped (Fig. 2C); outline is usually even. The influence on host cell is not pronounced.

Macrogametocytes (Fig. 2D-I, Table 2): The cytoplasm is homogenous in appearance and usually does not contain readily visible vacuoles; volutin granules are not seen. Gametocytes grow around the nuclei of infected erythrocytes, they slightly enclose the nuclei with their ends, but never encircle the nuclei completely, leaving a portion of the cytoplasm unoccupied by gametocytes (Fig. 2D-I). Advanced growing gametocytes adhere to the envelope of erythrocytes only by their ends (Fig. 2D, E). An unfilled space between the erythrocyte’s envelope and the gametocyte is present and readily visible at all stages of gametocyte growth. As a result, the growing and fully grown gametocytes assume a readily visible dumbbell-like shape, which is a characteristic feature of this species development (Fig. 2D-H). The largest fully grown gametocytes, however, were occasionally seen being nearly appressed to the erythrocyte envelope, resulting in a poorly visible space between the gametocytes and the envelope of erythrocytes, partly losing their dumbbell shape (Fig. 2I). Medium (Fig. 2F, G) and fully grown gametocytes (Fig. 2H, I) fill erythrocytes up to their poles. The parasite nucleus is compact, strictly subterminal in position, variable in form and is located close to the erythrocyte envelope. Pigment granules are roundish or oval, mostly of medium size (0.5–1 μm), usually randomly scattered throughout the cytoplasm. The outline of gametocytes is predominantly even. Nuclei of infected erythrocytes are slightly displaced laterally (Fig. 2D-I, Table 2).

Microgametocytes (Fig. 2J-P, Table 2): The general configuration is as for macrogametocytes, with the usual sexual dimorphism for haemosporidians, which are the pale staining of the cytoplasm, the large diffuse nuclei, and the grouping of pigment granules close to the gametocyte ends. Dumbbell-like shape is readily visible in growing gametocytes (Fig. 2J-L, N), but is often hardly visible in some fully grown gametocytes (Fig. 2O, P). Advanced non-dumbbell shaped microgametocytes (Fig. 2P) are more numerous than in macrogametocytes but remain the minority among all seen microgametocytes. The cytoplasm is usually paler stained at the gametocyte portion adhering to the erythrocyte envelope, when gametocytes were non-dumbbell shaped (Fig. 2P).

**Fig. 4:**
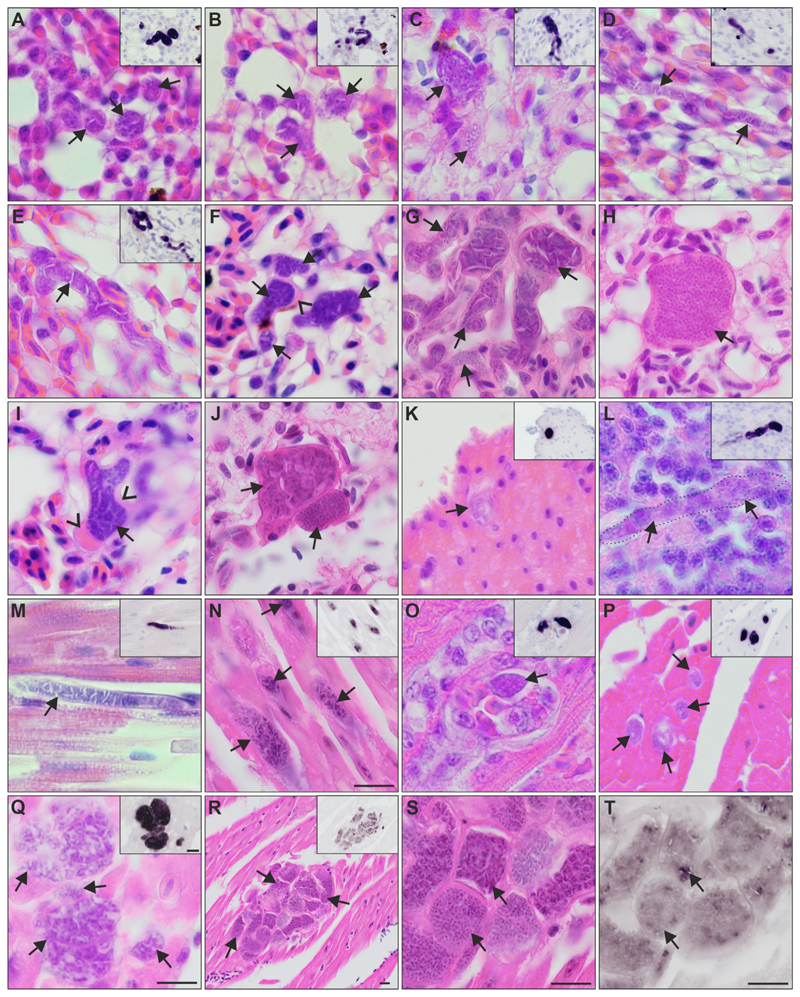
Meronts of *Haemoproteus dumbbellus* (lineage hEMCIR01) found in (A-J) the lungs, (K) gizzard, (L) brain, and (M-T) heart of two yellow hammers *Emberiza citrinella.* The meront generic origin in H & E-stained preparations was confirmed by chromogenic *in situ* hybridization (CISH) using a *Haemoproteus* genus-specific probe indicated by a purple signal in the insets of panels A-E and K-R. Independently of their maturation stage, meronts (indicated by arrows) differed greatly in shape, being round (A, B, K), oval (O, P) or elongate (D, L-N), and ranging in size from less than 10 μm (P) to more than 50 μm (D, E, M, Q). Elongate meronts in the heart appear to follow the shape of the muscle cells (M, N), whereas meronts in the lung tissue (E) were often of capillary shape. Meronts were surrounded by a thin eosinophilic wall, occasionally with bulges of unclear origin located at the periphery of nearly mature parasites (F, I). Note the development of angular-shaped cytomeres separated by clefts, which is a characteristic feature of meront maturation in this parasite species. Such clefts are particularly well visible between cytomeres before merozoite formation (E, G, J-upper arrow), and they disappear in mature meronts, which are overfilled with discrete merozoites (H, J-lower arrow). R-T show the same group of meronts at different magnifications. Note the difference in maturation among meronts (R-T), with mature, roundish merozoites, characterized by weak CISH signals (S, T, lower arrow), or the still developing cytomeres and stronger CISH signals (S, T, upper arrow). Arrowheads indicate the eosinophilic wall. Scale-bar = 10 μm.

Exo-erythrocytic stages (Fig. 4A-T): Exo-erythrocytic meronts were observed in the lungs, heart, gizzard, liver, leg muscle, and brain of infected birds, with the number of affected organs ranging among individuals from one to six (Table 1, Fig. 4). Among all individuals, the lungs were most commonly parasitized, while other organs were less often affected. In the lungs, numerous meronts were disseminated over the section and often clustered in small groups (Fig. 4F, G, J, N-S). The meronts varied in shape, size, and stage of maturation, indicating asynchronous development. They were either roundish or of irregular form (Fig. 4A, B, F-J, O-T), sometimes worm-like or branching meronts, following the shape of blood capillaries (Fig. 4L, C-E, M, N), suggesting localization in endothelial cells. Meront size, measured by the largest diameter, varied from 3 to 44 μm. In the heart, several meronts were located in cardiomyocytes. The meronts appeared solitary (Fig. 4M), in loose groups (Fig. 4N-P), or in juxtaposition with each other (Fig. 4R-T). Single meronts varied from 8 to 35 μm in their largest diameter. Solitary meronts were oval (Fig. 4O, P) or elongated (Fig. 4M, N), while adjoining meronts showed various shapes such as round, oval, cubic, or angular forms (Fig. 4R, S). In the muscular layer of the gizzard, only a few meronts were detected, which were roundish (Fig. 4K) or elongated and reached about 40 μm in length. In the brain of one individual, only a single elongated meront was observed (Fig. 4L). Meronts found in the leg muscles looked similar to meronts found in the heart, being mainly elongated.

Most detected meronts were growing meronts, showing different stages of development, independent of their size. Few early meronts were identified and characterized by an amorphous appearance with prominent cytoplasmic clefts but lacked recognizable developing merozoites, indicating that meronts were still developing (Fig. 4D, E, K). The majority of meronts showed more advanced stages of development with developing merozoites arranged in irregular, sometimes angular-shaped cytomeres and separated by cytoplasmic clefts (Fig. 4C). In growing meronts, cytomeres seemed to gradually disappear while developing merozoites became more conspicuous by the aggregation of nuclei (Fig. 4F, I, J, Q-T). Beside growing meronts, a few nearly mature meronts were identified. In these, cytomeres or clefts were invisible or barely visible as they contained numerous discrete merozoites of about 0.5 μm (Fig. 4H, J, S). Occasionally, mature meronts located in larger blood vessels, probably representing detached infected host cells or liberated meronts. Meronts were commonly covered by a thin eosinophilic wall. The nuclei of infected cells were rarely recognizable. Bulb-like eosinophilic bulges were occasionally observed at the periphery of nearly mature meronts (Fig. 4F, H, I). These bulges were similar in colour and refractivity to the observed wall around the meronts, but their origin was unclear.

No inflammatory reactions were associated with the detected meronts.

Remarks: Dumbbell-like shape of growing gametocytes is a common feature in avian *Haemoproteus* spp. due to the presence of unfilled space between the gametocytes and the envelope of erythrocytes (Valkiūnas, 2005). However, this space remains and is readily visible in the majority of fully grown gametocytes only in *H. dumbbellus* n. sp. This is a unique character of *H. dumbbellus*, which can be readily distinguished from other species of haemoproteids parasitizing passeriform birds based on this feature.

Histological sections subjected to CISH revealed focal exo-erythrocytic signals in the brain of one bird, but the parasite was not found in the corresponding H & E-stained section. In other words, meronts certainly develop in brain, but their intensity is probably low, as in most organs with exception of the heart and lungs. In most CISH sections, exo-erythrocytic signals were deep purple and easy to distinguish (Fig. 4A-E, K-Q). However, in the meronts that grouped tightly in the heart, CISH signals were less intense, corresponding to more mature meronts with developed merozoites (Fig. 4R, T).

### 3.4 *Haemoproteus hirundinis* (lineage hDELURB2): gametocytes and exo-erythrocytic development

Gametocytes present in the blood films of *D. urbicum* displayed characteristics of *H. hirundinis* parasite, including the main diagnostic characters of the species (Fig. 1A-F), such as the pattern of growth around the host cell nucleus, without encircling it completely (Fig. 1D, F); absence of growing dumbbell-shaped gametocytes (Fig. 1C); fully grown gametocytes filling the poles of the erythrocytes and adhering to both the envelope of the host cell and its nucleus (Fig. 1D, F); strictly subterminal position of nuclei in fully grown macrogametocytes (Fig. 1D); and variable size of pigment granules (Fig. 1D, F). This parasite morphology was the same as described before (Valkiūnas, 2005) and its detailed description was not repeated here.

**Fig. 5:**
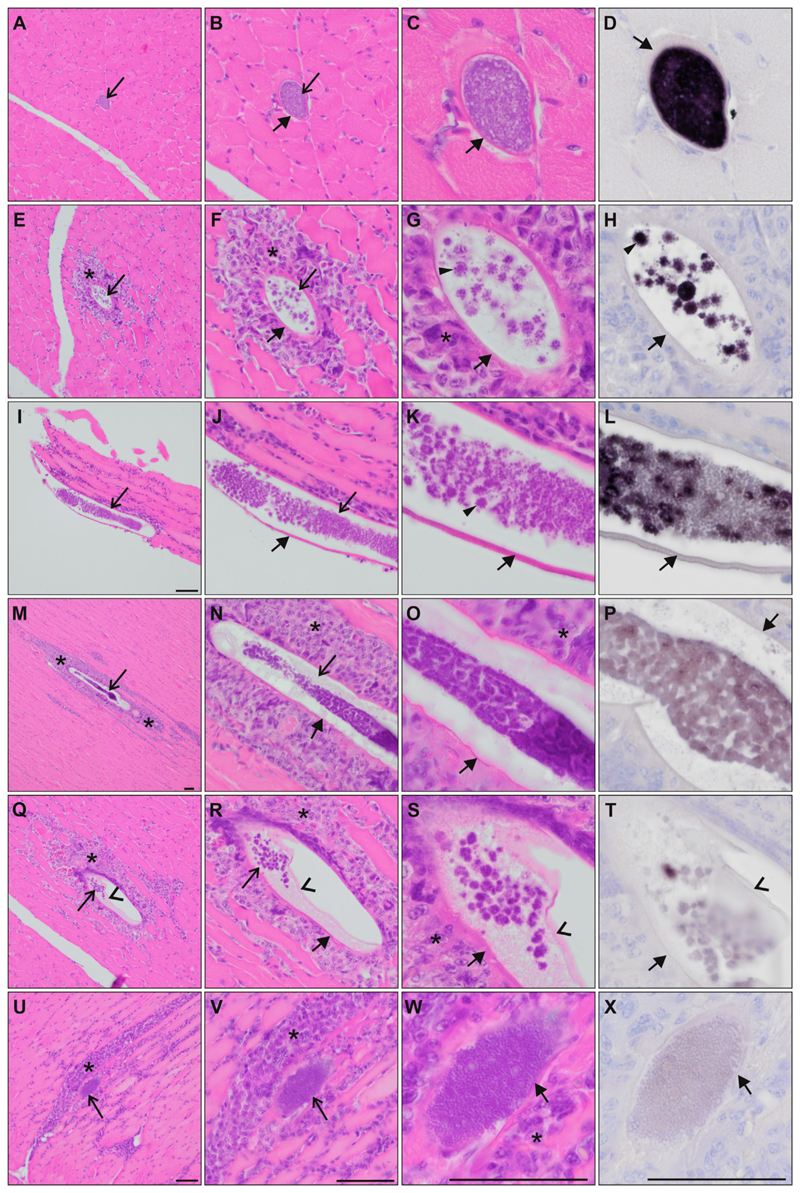
Megalomeronts of *Haemoproteus hirundinis* (lineage hDELURB2) found in the (A-X) pectoral muscles of two common house martins *Delichon urbicum.* The megalomeront generic origin in H & E-stained preparations was confirmed by chromogenic *in situ* hybridization (CISH) using a *Haemoproteus* genus-specific probe (purple signal) on a subsequent section (D, H, L, P, T, X). Megalomeronts (indicated by the longer arrows) were elongate, following the muscles cells. In transverse sections, they appeared as roundish or oval bodies (A-D). Megalomeronts were covered by a capsular-like wall of host origin (indicated by the shorter arrows). Note the presence of predominantly small roundish cytomeres, with merozoites budding-off at their periphery, giving maturing cytomeres various star-like appearances (indicated by the triangles in G, H, K). Note the differences in probe signal intensity among megalomeronts of different maturation, varying from strong signal in young megalomeronts (D, H) over less intense signal in maturing megalomeronts (L) to almost no visible signal in fully mature megalomeronts (P, T, X). Inflammatory host cell infiltration (indicated by the asterisks) was observed around megalomeronts (E-G, I-K, M-O, Q-S, U-W). Note the presence of a thin membrane partially detached from the inner wall of the megalomeront (indicated by the arrowheads in Q-T), and still covering the parasite. Mature megalomeronts were overfilled with discrete, roundish merozoites (W). Scale-bar = 50 μm.

Exo-erythrocytic stages were found only in the pectoral muscles of two out of six infected *D. urbicum.* The megalomeronts were mostly elongated (Fig. 5I-X), following the muscle cells, while in transversal sections, they appeared oval (Fig. 5A-H). They were all covered by an eosinophilic, capsular-like wall of host origin. The megalomeronts varied in size and maturity; the largest were up to 353 μm at their longest diameter. Young megalomeronts were characterized by a more or less homogenous, light basophilic content without recognizable cytomeres (Fig. 5A-C). Growing megalomeronts showed small roundish cytomeres, from which merozoites seemed to bud off at the periphery, giving the structures a star-like shape (Fig. 5G, H, K). Mature megalomeronts were packed with numerous discrete merozoites, cytomeres were not identifiable (Fig. 5U-W).

Remarks: Growing megalomeronts were characterized by intense CISH signals (Fig. 5D, H), whereas in more advanced or mature megalomeronts, CISH signals appeared less intense (Fig. 5P-X). One megalomeront presented individual cytomeres with developing and mature merozoites (Fig. 5I-L), which is reflected by varying intensities of the CISH signals within the megalomeront. In one megalomeront, an inner envelope seemed to have detached from the capsular-like wall (Fig. 5Q-S), but still covered the parasites; it was not stained by the Haemo18S probe (Fig. 5T).

Moderate to severe inflammatory lesions were observed around most maturing megalomeronts (Fig. 5E-G, M-O, Q-S, U-W).

Neohapantotypes (gametocytes, *D. urbicum*, sampled in 2021.05.19 Ventė Cape, Lithuania, coll. Mikas Ilgūnas, accessions nos. 49428NS, 49436NS, 49437NS; exo-erythrocytic stages: pectoral muscles, accessions nos. 49433NS, 49434NS, 49440NS, 49441NS, other data as for gametocytes) were deposited to the NRC. Neoparahapantotypes (gametocytes, *D*. *urbicum*, 2021.05.19, Vente Cape, Lithuania, coll. Mikas Ilgūnas, accessions nos. 49429NS, 49438NS, 49439NS; exo-erythrocytic stages: pectoral muscles accessions nos. 49435NS, 49442NS, other data as for gametocytes) were deposited to the NRC. Neoparahapantotypes (gametocytes, *D. urbicum*, 2021.05.19, Ventė Cape, Lithuania, coll. Mikas Ilgūnas, accessions nos. G466253, G466254, G466258; exo-erythrocytic stages: accessions nos. G466255 – G466257, G46259 – G466261, other data as for gametocytes) were deposited to the Queensland Museum, Queensland, Australia. Additional voucher preparations from *D. urbicum* sampled at the type locality: gametocytes (accession nos. 49430NS – 49432NS, 49443NS – 49445NS) were deposited to the NRC.

## 4 Discussion

The key results of the present study are i) the discovery and description of exo-erythrocytic stages in two haemoproteid species and ii) the newly described *H. dumbbellus* n. sp. (lineage hEMCIR01) in *E. citrinella* and the assignment of the lineage hDELURB2 to the species *H. hirundinis*, a parasite of common swallows and house martins (Hirundinidae).

*Haemoproteus dumbbellus* n. sp. differs from other *Haemoproteus* parasites parasitizing passeriform birds by the presence of a readily visible space between the parasite and the erythrocyte envelope in fully grown gametocytes, giving the gametocytes a distinct dumbbell-like shape even at final stage of growth in the blood (Fig. 2H). This parasite is prevalent in *E. citrinella* (Passeriformes), its type vertebrate host.

*Emberiza citrinella* is a resident bird in Europe (Shirihai and Svensson, 2018a), with molecular records of the lineage hEMCIR01 from two other European countries besides Lithuania and Austria: 30 individuals were positive in Sweden (Ellis et al., 2020), and one in Slovakia (Šujanová et al., 2021). According to the MalAvi database, the lineage hEMCIR01 was also found in one *E. citrinella* from the United Kingdom (Dunn et al., 2014), however, the GenBank accession number indicated in the paper refers to a sequence (hEMRUT01), which differs from hEMCIR01 by one nucleotide. *Emberiza cirlus*, another bird species from the Emberizidae family, is a native resident in Southern Europe and Northern Africa (Shirihai and Svensson, 2018b), with records of hEMCIR01 in two individuals: one from Bulgaria (Dimitrov et al., 2010), and one from Morocco (Mata et al., 2015). The record of hEMCIR01 in S. *nebouxii* is the only one from South America, but the first 56 nucleotides are missing from the barcoding sequence, with the remaining nucleotides matching hEMCIR01 (Levin et al., 2011); it is thus uncertain whether the parasite really is hEMCIR01. As this record originated from a bird of a distant order and a different continent as all other records, it was likely the result of a contaminated sample (see Bensch et al., 2021). The record in *Phylloscopus trochilus* (Phylloscopidae), a long-distance migrant from Africa breeding in Europe and Russia (Shirihai and Svensson, 2018b), is from Sweden, where infected *E. citrinella* were also reported (Ellis et al., 2020). It is unknown if this record resulted from a competent host and complete development (gametocytes should be present) or an abortive infection, with PCR amplification from circulating sporozoite stages or exo-erythrocytic merozoites. Based on these limited molecular data available, *H. dumbbellus* appears to be of European distribution and probably is specific to its type vertebrate host and closely related *Emberiza* species.

In the present study, the lineage hDELURB2 was linked to *H. hirundini*s, confirming earlier predictions that the lineage might belong to this morphospecies (Chagas et al., 2019). Previously, *H. hirundinis* was only linked to the lineage hDELURB1 (Valkiūnas et al., 2014), which differs from hDELURB2 by 7 bp or 1.5% in the 478 bp *cytb* gene barcoding region. According to the MalAvi database, both lineages were frequently found in *D. urbicum* sampled in Europe, with 371 records for hDELURB1 (out of 1101 birds tested) and 271 records for hDELURB2 (out of 1082 birds tested) (MalAvi database, last access in September 2022 (Bensch et al., 2009)), indicating a similar prevalence for both lineages (25%) in this bird species. Apart from *D. urbicum*, hDELURB2 was also found in two barn swallows *Hirundo rustica* (Von Rönn et al., 2015, Garcia-Longoria et al., 2019), and 10 sand martins *Riparia riparia* (Ciloglu et al., 2020; Hahn et al., 2021), including one record under the lineage name hRIPRIP07, which is identical to hDELURB2 over the 478 bp section.

This study discovered the exo-erythrocytic stages of *H. dumbbellus* and *H. hirundinis*. These are the first known reports of tissue stages of haemoproteids, which were identified to species levels and parasitizing birds of the families Hirundinidae and Emberizidae. Both parasite species appeared phylogenetically closer to species developing meronts, e. g. *H. attenuatus* hROBIN1 (Hernández-Lara et al., 2021), *H. passeris* (Peirce, 1976), than to species developing only megalomeronts, e. g. *H. minutus, H. majoris, H. pastoris, H. syrnii* (Himmel et al., 2019; Ilgūnas et al., 2019, 2022; Ortiz-Catedral et al., 2019; Duc et al., 2020, 2021). However, exo-erythrocytic stages of *H. dumbbellus* and *H. hirundinis* were readily different from each other. *Haemoproteus dumbbellus* developed only meronts (Fig. 4), whereas *H. hirundinis* developed only megalomeronts (Fig. 5). The two species also showed different sites of exo-erythrocytic development: *H. hirundinis* megalomeronts were found only in the pectoral muscles, whereas meronts of *H. dumbbellus* were found in diverse organs, including lungs, liver, gizzard, heart and leg muscles (Table 1). This could indicate a preference in the parasite development, with *H. hirundinis* developing in specialized muscle cells, and *H. dumbbellus* in non-specialised cells, which are present in many organs. It is interesting to note that *H. passeris* was placed in the same clade as *H. dumbbellus* and other meront-forming species (Fig. 3) but has been reported to develop both meronts (Peirce, 1976) and megalomeronts (Burtikashvili, 1978; Valkiūnas and Iezhova, 2017) in house sparrows *Passer domesticus*, although this was found in different localities and studies (UK, Georgia). It remains unclear if these two authors were dealing with same or different genetic lineage of *H. passeris*.

*Haemoproteus* spp. with reported megalomeronts were scattered throughout the phylogeny (Fig. 3) and often developed in several organs: *H. pastoris* in the intestine, kidneys, lungs, oesophagus, gizzard, brain, spleen and trachea (Duc et al., 2021); *H. majoris* in the kidneys, lungs, liver, spleen, and intestine (Ilgūnas et al., 2019; Duc et al., 2020); *H. minutus* in the heart and gizzard (Ortiz-Catedral et al., 2019); *H. asymmetricus* in the heart (Himmel et al., 2019); *H. passeris* in the lungs and liver (Valkiūnas and Iezhova, 2017); *Haemoproteus sacharovi* in the gizzard (Farmer, 1964) and *Haemoproteus velans* and *H. syrnii* in the muscles (Groff et al., 2019; Ilgūnas et al., 2022). Interestingly, megalomeronts of *H. sacharovi, H. velans*, and *H. syrnii* all developed in muscle tissues (smooth muscles for *H. sacharovi*) and cluster in the phylogeny (Fig. 3). However, megalomeronts of *H. hirundinis*, also found exclusively in the pectoral muscles of its host, did not cluster with them. Megalomeront morphology is also quite different in all investigated species, with *H. hirundinis* being slender and of a smaller size compared with the megalomeronts found in the other three species (Farmer, 1964; Groff et al., 2019; Ilgūnas et al., 2022). Unfortunately, there is still no data available on the exo-erythrocytic development of species that do cluster more closely with *H. hirundinis* (Fig. 3), so any generalization about the morphology and site of megalomeronts development remain premature based of available phylogenetic data.

For hDELURB1, a lineage previously attributed to *H. hirundinis*, data on exo-erythrocytic stages are absent. Due to the genetic similarity of the lineages hDELURB2 and hDELURB1, it is possible to presume that the latter would also develop megalomeronts in the pectoral muscles, analogous to a pattern observed in three *H. majoris* lineages, which all developed megalomeronts of similar morphology and location in different avian hosts (Ilgūnas et al., 2019; Duc et al., 2020). Further studies are needed to test this hypothesis.

*Haemoproteus* parasites were previously often neglected in veterinary medicine as thought to be relatively harmless to their avian hosts (Bennett et al., 1993). However, the recent reports of death of non-adapted birds due to molecularly proven haemoproteosis brought back interests on the study of the parasite exo-erythrocytic stages, which might markedly damage various organs (Ortiz-Catedral et al., 2019). A study recently reported cell necrosis associated with the development of *Haemoproteus* megalomeronts (Ilgūnas et al., 2022), while in the present study, inflammatory lesions were observed around the megalomeronts found in *D. urbicum*, but not around the small meronts found in *E. citrinella.* This could be due to their difference in size, inducing different host reactions or by the different stages of maturation of meronts/megalomeronts. When megalomeronts burst and release merozoites, the parasites are no longer protected by the capsular-like wall and become exposed to the hosts immune system, which can elicit an inflammatory response. The findings of this study demonstrate that natural hosts can be affected by haemoproteosis due to inflammatory reactions induced by exo-erythrocytic stages, which can be severe during excessive multiplication of the parasites. This calls for more attention to these pathogens and their veterinary importance not only in poultry and pet birds, but also in wildlife.

The markedly variable shape and small size of meronts makes them difficult to recognize in H & E-stained histological sections, as for example, in the case of the meront observed in the brain of *E. citrinella* (Fig. 4L). *Haemoproteus* meronts can sometimes appear outwardly similar to phanerozoites of some *Plasmodium* parasites (Valkiūnas, 2005). The CISH technique helps to locate such meronts and to confirm their generic origin using genus-specific probes, which target the *18S* ribosomal RNA of the parasites (Himmel et al., 2019). This method also provides insights into RNA expression of the parasites during the development of meronts and megalomeronts, as the intensity of the CISH signals should reflect the abundance of RNA molecules (Himmel et al., 2019). For example, the parasites should express more RNA as they actively multiply, and less RNA upon complete maturation leading to varying signal intensities among stages of different maturation. This pattern was well observed in megalomeronts of *H. hirundinis* (Fig. 5D, H, L, P, T, X), where young or growing megalomeronts showed deep dark CISH signals (Fig. 5D, H, L) and megalomeronts containing mature merozoites showed little CISH signals (Fig. 5X). One megalomeront also showed both darker (developing cytomeres) and less intense (mature merozoites) signals at the same time indicating asynchronous maturation of cytomeres and merozoites (Fig. 5L). A similar pattern was also observed in meronts of *H. dumbbellus* found in the heart muscle (Fig. 4T).

It is still unclear, whether a host will contain more exo-erythrocytic stages during high or low parasitaemia. In other words, it is unclear if high parasitemia is always accompanied with intensive exo-erythrocytic development. This information would be important for the decision process on whether, and at which time of infection to sacrifice infected birds aiming to investigate exo-erythrocytic development of the parasites. Among the four *E. citrinella* with 0.35% to 1.04% parasitaemia, the individual with the lowest infection intensity (0.35%) did not show any meronts in the sections analysed, while all the other individuals (0.69%, 0.98%, and 1.04% parasitaemia) exhibited meronts in diverse organs (Table 1). Among the six *D. urbicum* with 0.04% to 0.53% parasitaemia (Table 1), two individuals (0.34% and 0.53% parasitaemia) showed megalomeronts, but in the other two individuals with comparable parasitaemia (0.51% and 0.53%), the exo-erythrocytic stages were absent in the investigated sections (Table 1). A previous study on *H. pastoris* exo-erythrocytic development also reported the presence of megalomeronts during different parasitaemia (from 1% to 26%), except in one individual with a parasitaemia of 10% (Duc et al., 2021). Based on these data, no pattern emerges as to which parasitaemia intensity would be optimal for the investigation of exo-erythrocytic development in the avian host. Lower parasitaemia could imply ongoing exo-erythrocytic development with presence of mainly immature meronts and thus few infected erythrocytes yet, whereas higher parasitaemia could imply advanced exo-erythrocytic development with bursting meronts or being on the verge to burst (merozoites are released and infect erythrocytes). However, considering that meronts of different maturity were observed within the same individuals (Fig. 4) – a pattern also observed in megalomeronts of other species (Himmel et al., 2019; Ortiz-Catedral et al., 2019; Duc et al., 2021) and suggesting asynchronous merogony – the intensity of parasitaemia might not necessarily correlate with the abundance of merogonic stages in the tissues. In other words, the level of parasitaemia is likely a poor indicator of whether to find exo-erythrocytic stages in the organ of infected birds. Selection of birds with single infections is most important during fieldwork as it markedly simplifies further parasite species identification using general molecular primers and characterization of the developmental pattern of a single parasite.

The identification of haemosporidian species requires observation of gametocytes in blood films, and the detection of their exo-erythrocytic stages in tissues depends on histological work; both techniques require microscopic examinations. The specific parasite lineage is determined by molecular methods (PCR, DNA analysis). Due to the remarkable morphological diversity of tissue stages in haemosporidian parasites (Valkiūnas and Iezhova, 2017), the application of CISH is very helpful to prove generic identity of reported tissue stages. The combination of all these methods allows a more complete and accurate characterization of individual infections (Valkiūnas et al., 2006; Bensch and Hellgren, 2020). With about 177 species of *Haemoproteus* described (Valkiūnas and Iezhova, 2022), microscopic studies rely on experience and training to be able to identify the parasites (Valkiūnas, 2005; Valkiūnas and Iezhova, 2018, 2022); while molecular studies are relatively easy to apply and only require general to specific primers and PCR for amplification of the parasites’ DNA and determination of lineages. Of about 1900 *Haemoproteus* lineages recorded so far, only 159 have been assigned to their corresponding morphospecies (MalAvi database, last access in September 2022, (Bensch et al., 2009)), leaving a large number of unlinked lineages and potentially new species. As most published studies do not investigate blood films, it is difficult to know whether a positive PCR amplification comes from the successfully developed parasites in their avian hosts (i.e. gametocytes developed), or from the injected sporozoites, which could not initiate infection or initiate only a partial development, resulting in the presence of incompletely developed exo-erythrocytic meronts and their remnants in the circulation (Valkiūnas and Iezhova, 2017). On the other hand, a negative molecular result could imply the absence of infection (no gametocytes, sporozoites or remnants of tissue stages), or result from mismatches between the primers and the parasite DNA. For example, we observed gametocytes in the blood films of six *D. urbicum*, but only two individuals were positive by PCR with the primers HAEMNFI/HAEMNR3 - HAEMF/HAEMR2 (Bensch et al., 2000; Hellgren et al., 2004). However, when using the primers PLAS1F/HAEMNR3 and 3760F/HAEMJR4 (Beadell et al., 2004; Hellgren et al., 2004; Duval et al., 2007; Pérez-Rodríguez et al., 2013), all samples were positive. Comparing the primer sequences HAEMF/HAEMR2 to a longer sequence of hDELURB2 previously submitted (MK843311 (Chagas et al., 2019)), three mismatches were found for both the forward and reverse primers, which could explain lower primer affinity and amplification rate. Thus, alternative primers are recommended for screening of *D. urbicum* or bird species infected with closely related parasite species. Ultimately, both molecular and microscopic methods are crucial for gaining a more complete picture of the lineage diversity present in a given bird species and for the discovery of new lineages and parasite species.

In summary, this study contributes to the knowledge of exo-erythrocytic development of avian Haemoproteidae species due to the discovery of the tissue stage in two *Haemoproteus* parasites – *H. dumbbellus* n. sp. and *H. hirundinis.* The new species, *H. dumbbellus*, and its exo-erythrocytic stages were described from *E. citrinella* infected with the lineage hEMCIR01; only meronts of markedly different shapes were observed in the lungs, heart, brain, liver, leg muscles and gizzard. The lineage hDELURB2 was attributed to *H. hirundinis* observed in *D. urbicum*, and its exo-erythrocytic stages – megalomeronts – were only found in the pectoral muscles. The megalomeronts were of unique morphology among avian haemoproteids due to the star-like appearance of developing cytomeres. This study highlights the remarkable diversity of exo-erythrocytic stages throughout *Haemoproteus* spp., which develop meronts or megalomeronts and infect one or several organs. Analysis of the available data suggest that the exclusive development of meronts might be restricted to fewer *Haemoproteus* species than previously thought, while megalomeronts seem to develop in more parasite species and probably is the predominate stage during exo-erythrocytic development of avian haemoproteids. Future investigations of other common *Haemoproteus* species are needed to examine which exo-erythrocytic stage (meront, megalomeront, or both) in which parasite species might predominantly occur, and whether molecular phylogenies can be used in practical parasitological work to predict patterns of exo-erythrocytic development using simply DNA sequence information.

## Figures and Tables

**Table 1:** Molecular and morphological identification of parasites detected with location of their exo-erythrocytic stages in *Emberiza citrinella* and *Delichon urbicum*.

Individual	Host species	Collection date	Origin	Parasitaemia (%)	*cytb* lineage	Parasite species	Location (and type) of exo-erythrocytic stages
AH0608	*Emberiza citrinella*	2004-08-17	Austria	n/a	hEMCIR01	*Haemoproteus dumbbellus*	Lungs, gizzard (meronts)
AH0611	*E. citrinella*	2004-08-17	Austria	n/a	hEMCIR01	*H. dumbbellus*	Heart, lungs (meronts)
556/17R	*E. citrinella*	2017-05-15	Lithuania	0.54	hEMCIR01	*H. dumbbellus*	Not available
579/17R	*E. citrinella*	2017-05-16	Lithuania	0.26	hEMCIR01	*H. dumbbellus*	Not available
19/18R	*E. citrinella*	2018-05-16	Lithuania	0.69	hEMCIR01	*H. dumbbellus*	Lungs (meronts)
305/19R	*E. citrinella*	2019-05-11	Lithuania	0.35	hEMCIR01	*H. dumbbellus*	Not found
242/21R	*E. citrinella*	2021-05-17	Lithuania	0.98	hEMCIR01	*H. dumbbellus*	Heart, liver, lungs, leg muscle, gizzard (meronts)
349/21R	*E. citrinella*	2021-05-19	Lithuania	1.04	hEMCIR01	*H. dumbbellus*	Heart, liver, lungs, leg muscle, gizzard, brain (meronts)
337/17R	*Delichon urbicum*	2017-05-09	Lithuania	0.04	hDELURB2	*Haemoproteus hirundinis*	Not found
79/18R	*D. urbicum*	2018-05-18	Lithuania	0.2	hDELURB2	*H. hirundinis*	Not found
82/18R	*D. urbicum*	2018-05-18	Lithuania	0.51	hDELURB2	*H. hirundinis*	Not found
329/21R	*D. urbicum*	2021-05-19	Lithuania	0.53	hDELURB2	*H. hirundinis*	Pectoral muscle (megalomeronts)
323/21R	*D. urbicum*	2021-05-19	Lithuania	0.34	hDELURB2	*H. hirundinis*	Pectoral muscle (megalomeronts)
340/21R	*D. urbicum*	2021-05-19	Lithuania	0.53	hDELURB2	*H. hirundinis*	Not found

*Cytb*, cytochrome b gene; n/a, not available.

**Table 2:** Morphometry of host cells and fully grown gametocytes of *Haemoproteus dumbbellus* n. sp. (lineage hEMCIR01) from the blood of the yellowhammer *Emberiza citrinella.* Measurements for length and width are in micrometres (μm), and area is μm^2^.

Features	Measurement (μm) (mean ± S.D.)
**Uninfected erythrocytes**	
Length	10.6–12.5 (11.4 ± 0.3)
Width	5.7–7.0 (6.4 ± 0.1)
Area	48.9–66.0 (57.2 ± 20.3)
**Uninfected erythrocytes nucleus**	
Length	4.7–6.0 (5.3 ± 0.1)
Width	2.0–2.7 (2.3 ± 0.03)
Area	8.0–13.0 (10.1 ± 1.3)
**Macrogametocytes**	
**Infected erythrocyte**	
Length	10.7–13.1 (12.0 ± 0.3)
Width	5.7–7.9 (7.1 ± 0.3)
Area	57.8–76.9 (66.8 ± 28.4)
**Infected erythrocyte nucleus**	
Length	4.6–6.5 (5.3 ± 0.2)
Width	2.1–2.8 (2.4 ± 0.03)
Area	8.6–12.1 (10.4 ± 0.9)
**Gametocyte**	
Length	13.6–17.2 (15.4 ± 1.0)
Width	2.3–4.1 (3.4 ± 0.2)
Area	37.6–50.9 (43.4 ± 14.8)
**Gametocyte nucleus**	
Length	2.1–3.0 (2.5 ± 0.1)
Width	1.2–2.5 (1.9 ± 0.1)
Area	2.3–5.9 (4.1 ± 0.8)
**Pigment granules**	13.0–17.0 (14.5 ±2.0)
**NDR**	0.5–0.9 (0.7 ± 0.02)
**Microgametocytes**	
**Infected erythrocyte**	
Length	11.5–13.9 (12.3 ± 0.3)
Width	6.1–8.4 (7.3 ± 0.2)
Area	61.6–79.1 (69.4–24.4)
**Infected erythrocyte nucleus**	
Length	1.9–5.7 (5.0 ± 0.6)
Width	2.0–2.5 (2.3 ± 0.02)
Area	8.7–11.0 (9.8 ± 0.5)
**Gametocyte**	
Length	13.7–19.1 (17.1 ± 2.0)
Width	2.7–4.5 (3.7 ± 0.2)
Area	36.7–58.8 (47.9 ± 30.0)
**Gametocyte nucleus**	
Length	5.7–10.7 (8.6 ± 1.6)
Width	1.8–7.1 (3.0 ± 1.2)
Area	16.6–30.7 (23.6 ± 15.4)
**Pigment granules**	n/a
**NDR**	0.6–0.9 (0.7 ± 0.01)

n/a, no information is available as pigment granules were predominantly grouped (Fig. 2K-P) and difficult to count; NDR, Nuclear displacement ratio according to Bennett and Campbell (1972).
